# Supplementary material for: Application of a text mining method in navigation and communication for enhancing maritime safety
Source: PLoS One. 2024 Mar 22;19(3):e0299582. doi: 10.1371/journal.pone.0299582 (PMC10959359; doi:10.1371/journal.pone.0299582)
Supplement: Attachment — Submitted filename: Review comments - Authors.docx [file pone.0299582.s002.docx]

Dear Author(s),

Topic of the article is interesting. However, following comments should be addressed prior to further processing of the article.

1) Refer to whole article: Similarity is very high i.e. 32%
2) Refer to whole article: Too many formatting issues.
3) Refer to whole article: Authors have claimed that auto generated ontology will lead to safe sea communication. What do they think about machine error?
4) Refer to whole article: Language grammars are usually very complicated. Which language is targeted by the authors and how do they address grammar issue which may lead to communication of false signal?
5) Refer to Whole article: Scope of this study seems limited as the proposed model focuses on a single language. What about communication across different languages?
6) Refer to figure 2 & 3: Article is written in English however another language is used in figures.
7) Refer to sub-section 1.2.3: Authors need to include a flow chart for easier understanding of the process to the novice reader.
8) Refer to all figures: Figures quality is very low.
9) Refer to table 2: Content is replicated in table 2 and figure 5. Further, most of the references are very old. No reference from 2023. Authors need to include recent references.

Good luck.
