# Supplementary material for: Application of a text mining method in navigation and communication for enhancing maritime safety
Source: PLoS One. 2024 Mar 22;19(3):e0299582. doi: 10.1371/journal.pone.0299582 (PMC10959359; doi:10.1371/journal.pone.0299582)
Supplement: Attachment — Submitted filename: Response to Reviewers.docx [file pone.0299582.s003.docx]

**RESPONSE to REVIEWERS**

**Dear Reviewers,**

**thank you very much for your thorough substantive assessment**

**and editorial of our article. Thank you for your positive opinions as well as the critical comments. They constitute important tips to improve the quality of our research work. Below we provide answers to the questions asked and responses to the comments included in the review.**

1) **Refer to whole article: Similarity is very high i.e. 32%**

This article is a continuation of research on automatic communication in maritime transport. The authors continue their original research topic. There is little similarity between the activities in the studies. But this article introduces something new - the text mining method

2) **Refer to whole article: Too many formatting issues.**

Thank you for this attention. The article has been corrected

3) **Refer to whole article: Authors have claimed that auto generated ontology will lead to safe sea communication. What do they think about machine error?**

The phrase "machine error" can be understood as communication errors, user errors, calculation errors. The proposed communication model takes into account the occurrence of the mentioned errors. The authors state that machine errors may occur. Occurring in unusual, unpredictable and typical situations. These errors can come in two forms. The first one is – machine and computational errors. The second one is for processing errors: errors regarding data or numerical quantities.

With current research, the explanation for the above errors is non-existent. However, the authors do not deny that this is impossible.

4) **Refer to whole article: Language grammars are usually very complicated. Which language is targeted by the authors and how do they address grammar issue which may lead to communication of false signal?**

The analysis of maritime case law indicates that in the event of a lack of connection between a voice call and a second call, this was one of the basic charges against the ships that took part in the occurrence. Decision errors may be caused by failure to initiate voice communication, its effects, or misunderstanding of the information conveyed in this way. These errors may be related to stress, which in turn affects the use of mental control and personal safety, self-assessment scores and situational awareness, disturbance of characteristic features, prolongation of decision duration. Errors may also be missing when it comes to using the English language. Disadvantages are determined in an oral form, including: problems with decoding the message at the semantic level, polarization (tendency to express extreme opinions), labeling (noticing problems by naming them, rather than analyzing them), mixing facts and occurrence as well as static assessment ( i.e. lack of verification of opinions regarding changes in elements of reality).

The primary task of navigation is to ensure safe navigation by avoiding dangers during a sea voyage. The goal of establishing direct communication between ships and automating communication processes can reduce wrong decisions and, as a result, wrong actions resulting in maritime accidents. This mainly concerns dangerous situations requiring decisive action to avoid a collision, in particular excessive approach situations (a situation in which avoiding a collision requires concerted action by the navigators of the meeting ships).

Currently, communication at sea is based on conventional requirements regarding ship equipment and crew training. At the level of the international convention, rules for the law of the sea route (COLREGs) have also been established. Detailed communication procedures are specified in the International Radio Regulations issued by the ITU (International Telecommunication Union). However, the communication process itself requires a broad analysis of the situation based on existing procedures, as well as ship equipment and systems.

The MPDM regulations are designed to safely conduct maneuvers, e.g. passing, overtaking, and especially anti-collision maneuvers - without the use of voice and/or radio-electronic communication. Maneuvering and warning signals (light and sound) are permitted for ships that are mutually visible. However, ambiguities and discrepancies in the interpretation of terms such as "safe distance", "early enough", "change course to the right" or the interpretation of the meeting situation (e.g. overtaking or crossing courses), introduced the practice of correspondence, most often by phone, between navigators meeting ships.

The communication process is divided into individual elements: the sender, i.e. the initiator, the recipient and the message. The person initiating communication performs coding, i.e. transforming thoughts into words that he thinks will be understandable to the recipient, thus creating a message. Then comes the communication channel, i.e. the way in which the content is to be conveyed. The message reaches the said recipient. Decoding, i.e. the interpretation of the received information, depends on its perceptual capabilities.

In such a situation, a problem arises - the communication process understood in the field of maritime transport must be precisely defined. Its main task is to convey the message from the sender in such a way that it is clearly understood by the recipient. A large amount of information and the diversity of its types and scopes result in the need to process, integrate and select it. And most importantly – a clear form and interpretation. An example of ambiguity that a navigator may understand in different ways is: heave up the line/send heaving line.

The article presents sample dialogues in scenarios and the recording of messages in ontological notation - in English. The reason is that English is considered the basic language in maritime navigation.

There are current communication system procedures recommended by the ITU (International Telecommunications Union), but they are complicated and difficult to assimilate by operators.

The current state of knowledge confirms that all previous research in the field of maritime transport does not indicate a clear and working communication system based on ontology.

The use of ontology and the beginning of operating on semantic models contributes to obtaining new possibilities related to the collection and processing of information.

5) **Refer to Whole article: Scope of this study seems limited as the proposed model focuses on a single language. What about communication across different languages?**

Due to maritime law, which requires communication in English, the authors deal only with this language.

6) **Refer to figure 2 & 3: Article is written in English however another language is used in figures.**

Thank you for this attention. The drawings have been corrected

7) **Refer to sub-section 1.2.3: Authors need to include a flow chart for easier understanding of the process to the novice reader.**

Thank you for your attention. We added information about the process in the loop method

8) **Refer to all figures: Figures quality is very low.**

Thank you for your attention. The drawings have been corrected

9) **Refer to table 2: Content is replicated in table 2 and figure 5. Further, most of the references are very old. No reference from 2023. Authors need to include recent references.**

Thank you for your attention. The drawings and tabels have been corrected

The authors use the Statistical Yearbook of Maritime Economy 2022.

The provided data was included in the article.

In January 20234 - data from 2022 will be available.

This chapter contains information on accidents at sea to ships of Polish nationality, and marine salvage. 2. Data on accidents at sea cover ships of either Polish or non-Polish nationality when the incident or accident at sea occurred in Polish internal waters or Polish territorial sea. Passenger ro-ro ferries or highspeed passenger ships are also included when the incident or accident occurred outside the internal waters or territorial sea of the EU member state provided that the last port of call of that ship was a Polish Republic's seaport. In addition these statistics cover ships of gross tonnage below 50, i.e. fishing boats, yachts or tugs

10) **The simulation validation process in the manuscript is deemed too simplistic. In real maritime communication scenarios, numerous pronunciation and technical issues are prevalent, yet these challenges are not adequately reflected in the simulated settings. A more comprehensive simulation that mimics the complexities of actual maritime communication should be considered.
    The manuscript requires substantial revision, focusing on improving its formatting, enhancing the clarity of figures, and conducting a more realistic simulation validation. Addressing these issues will significantly enhance the overall quality of the manuscript.**

Three encounter situations and two vessel models (non-autonomous, real-time) were selected for the study:

- crossing courses: ship A on course 270^o^ , and ship B on course 000^o^ ; the meeting ships are of medium size (length ca. 170 m), both proceeding full ahead, in sight if each other;

- reciprocal or almost reciprocal courses, ship A on course 180^o^, ship B on course 000^o^; the meeting ships are of medium size (length ca. 170 m), both proceeding at full ahead and seeing each other;
- encounter with a vessel on relative bearing 112^o^ : vessel A proceeds on course 312^o^ , and vessel B on course 000^o^ ; the meeting vessels are of medium size (length approx. 170 m), both proceeding full ahead and sea each other.

The selected scenarios are given as standard ship meeting situations as described in the COLREGs. Nevertheless, they raise many interpretative doubts about the safety of the manoeuvre being performed.

The article presents a scenario of a collision situation of an encounter with relative bearing of 112^o^, because similar situations are often misinterpreted, and one of two rules are taken as applicable: overtaking right-of-way or intersecting courses.

The authors conducted simulations of all types of meetings - more and less complex.

Below we present sample simulations - not included in the article.

**Scenario one:** Ship A (heading 270^o^) sent a message to ship B (heading 000^o^) with information that it had the right of way and that the project was his responsibility (Fig. 1). Ship B was dispatched, but sent one more message in the form of information: that there was a navigational hazard on its right side and the ship away from ship A, crossing ahead of its bow. Ship A arrived and a request was sent for ship B to move slightly to the left (Fig. 2, Fig. 3). Ship B confirmed and changed course to port by 30^o^. CPA was 0.30 Mm. Send ship A in the form of information that it is giving more space to ship B and has sent your intention explaining that your course has been changed (40°) to the left. The trajectory shown in Fig. 4.

Table 1. Communication scenario (category: negotiations) with an ontological record for a meeting situation on intersecting courses

| Scenario  communication: negotiations | Ontology-based communication | Communication using natural language (Text mining) |
| --- | --- | --- |
| A to B: I have the right of way. | {ontology of communication, message structures, body, Tell, T_information}, {ontology of navigation, identification of situation, priority, I_have},  {ontology of communication, message structures, body, Tell, T_demand}, {ontology of navigation, identification of situation, give way} | I have the right of way. |
| B to A: yes, that's right, but I have a navigation hazard on the right. I have to step in front of you | {ontology of communication, phrase, roger}.  {ontology of communication, message structures, body, Answer, A_information},  {ontology of navigation, enviroments information, geographic information, navigation infrastructure, Navigational danger right}.  {ontology of communication, message structures, body, Tell, T_demand}, {ontology of navigation, features information, Ship maneuvering, Passing ,ahead of you}. | I confirm. I have a navigation hazard on the right side. I have to step in front of you. |
| A to B: OK, but change course a little to the left | {ontology of communication, phrase, roger}.  {ontology of communication, message structures, body, Tell, T_request}. {ontology of navigation, features information, navigation information, course, alter course, to port} | I confirm. Change course a little to the left. |
| B to A: OK | {ontology of communication, phrase, roger}. | I confirm. |
| A to B: to have more space, I will change course to the left | {ontology of communication, message structures, body, Answer, A_information},  {ontology of navigation, Features information, Ship manoeuvring, more space}. {ontology of communication, message structures, body Tell, T_intention}, {ontology of navigation, features information, navigation information, course, alter course, to port}. | I need more space. I'll change course to the left. |

source: authors’ work

Fig. 1. Scenario: intersecting courses, negotiations. Initial situation

source:: Transas ECDIS NaviSailor 3000i

Fig. 2. Scenario: intersecting courses, negotiations. Situation during the maneuver

source: Transas ECDIS NaviSailor 3000i

Fig. 3. Scenario: intersecting courses, negotiations. View from the bridge of the ship having the right of way

source:: Transas NaviTrainer 4000

Fig. 4. Actual trajectories for a meeting situation on intersecting courses (scenario: negotiations)

source: authors’ work

**Scenariusz 2**:	Ship B course: 000^o^, ship A 180^o^. Good visibility. Ship A informs ship B that they are almost straight ahead and proposes to change course to the right (Fig. 5). Ship B rejects this offer, sending a reply saying it is safe. Ship B proposes to pass each other on their starboard sides and maintain course and speed. Ship A agrees and sends a confirmation. Ship B also sends an acknowledgment. The trajectory shown in Fig. 6.

Table 2. Communication scenario (category: negotiations) with an ontological record for the situation of meeting ships on almost direct courses

| Scenario  communication: negotiations | Ontology-based communication | Communication using natural language (Text mining) |
| --- | --- | --- |
| A to B: we are heading almost straight, let's both change course to the right | {ontology of communication, message structures, body, Tell, T_information},  {ontology of navigation, features information, navigation information, course, head on}, {ontology of navigation, features information, navigation information, course, alter course, both to starboard} | We're heading almost straight, let's both change course to the right. |
| B to A: no, there are safe courses (i.e. it is safe), we are passing each other on the starboard sides, keep the course and speed | {ontology of communication, phrase, negativ},  {ontology of communication, message structures, body, Answer, A_informtion},  {ontology of navigation, features information, navigation information, course, safe course} {ontology of navigation, features information, Ship maneuvering, passing, starboard to starboard}, {ontology of navigation, identification of situation, keep_the_course_and_speed}. | . NO. There are safe courses, we are passing each other on the right sides, keep your course and speed |
| A to B: OK | {ontology of communication, phrase, roger} | I confirm |
| B to A: OK | {ontology of communication, phrase, OK} | OK |

source: authors’ work

Fig 5. Scenario: almost straight ahead, information. Situation when ships pass each other

source: Transas ECDIS NaviSailor 3000i

Fig. 6. Actual trajectories for encountering ships on nearly straight courses (scenario: information)
source: authors’ work
