# Supplementary material for: Application of a text mining method in navigation and communication for enhancing maritime safety
Source: PLoS One. 2024 Mar 22;19(3):e0299582. doi: 10.1371/journal.pone.0299582 (PMC10959359; doi:10.1371/journal.pone.0299582)
Supplement: Attachment — Submitted filename: Review comments - Authors.docx [file pone.0299582.s004.docx]

Dear Author(s),

My comments are addressed and I have no more comments.

Good luck.
