# Supplementary material for: Application of a text mining method in navigation and communication for enhancing maritime safety
Source: PLoS One. 2024 Mar 22;19(3):e0299582. doi: 10.1371/journal.pone.0299582 (PMC10959359; doi:10.1371/journal.pone.0299582)
Supplement: Attachment — Submitted filename: Response to Reviewers.docx [file pone.0299582.s005.docx]

Dear Reviewer,

Thank you very much for your further comments to our article. To ensure the quality of our research work is as good as possible, we provide the answer to the question below.

Data validation is a solution that uses research with the efficiency of empirical data. We try to ensure that the data of the congregations in our research are thorough and detailed. Validation is important because if it is false or incomplete, data may be exposed and incorrect decisions may be made.

Proposed models for controlling commands from the captain's bridge to the autonomous system and automatically launched using empirical data from the presented test scenarios. Due to the operation of the first model - the article as a concept added to semi-autonomous maritime communication.
The target models will be tested based on a significant data set, where:

- for the first model, the input data will be a set of sequences of events in the ontology, while the output will be sentences in a natural language;

- for the second model, the input will be commands in a natural language, and the output will be a sequence of events in the ontology.

The test method will use developed models for each pair of elements from the set, where the input to the model will be input data, and the generated output data from the models will be compared with the collected output data (correct data). In this way, it will be possible to compare the correctness of the results and determine the accuracy coefficients of the method. Work on the proposed models is aimed at achieving the highest possible accuracy based on the collected data set.

Data analysis software is an indispensable tool in empirical research. The authors plan to conduct advanced statistical analyses, which will enable a better understanding of the collected data and drawing accurate conclusions. This is the direction of further research work by the authors.
